# Clinical outcomes and survival benefits of craniotomy in breast cancer patients with brain metastases: Focusing on candidate selection and early mortality

**DOI:** 10.1016/j.bas.2026.106158

**Published:** 2026-07-07

**Authors:** Soo Bin Lee, Seo Yeon Kim, Sung Jun Ahn, Bio Joo, Hun Ho Park, Jaejoon Lim, Jiwoong Oh, Jihwan Yoo

**Affiliations:** a Department of Neurosurgery, Brain Tumor Center, Gangnam Severance Hospital, Yonsei University, College of Medicine, Seoul, Republic of Korea; b Department of Radiology, Gangnam Severance Hospital, Yonsei University, College of Medicine, Seoul, Republic of Korea; c Department of Neurosurgery, Bundang CHA Medical Center, CHA University College of Medicine, Seongnam, Republic of Korea; d Department of Neurosurgery, Severance Hospital, Yonsei University, College of Medicine, Seoul, Republic of Korea

**Keywords:** Breast cancer, Brain metastases, Craniotomy, Decision-making, Triple negative breast cancer

## Abstract

**Background:**

Surgical decision-making for breast cancer (BC) brain metastases (BM) remains challenging due to surgery-related mortality risk. This study evaluated survival benefits of craniotomy and investigated causes of early postoperative mortality to refine surgical candidate selection.

**Methods:**

This retrospective cohort study included 157 BC patients with BM (July 2006–August 2022). Demographics, cancer subtype, performance score, BM number, extracranial metastasis, and treatment details were collected. Survival was analyzed using Kaplan–Meier and Cox regression models. Propensity score matching (PSM) was applied to control for confounding. A subgroup analysis of “short survivors” (survival <6 months) investigated early mortality causes.

**Results:**

The mean age was 52.4 years; 36.9% had triple-negative breast cancer and 21.7% were HER2-positive. Craniotomy was performed in 37.4% of patients and was associated with improved overall survival (30-month survival rate: 30%, p < 0.001). Multivariate analysis identified oligometastases (p < 0.001), multiple metastases (p = 0.007), and absence of radiotherapy exposure (p = 0.005) as independent predictors of poor prognosis. Among 16 short-term survivors (<6 months), only three (18.8%) died within 30 days due to surgery-related complications; the remainder died from systemic disease progression.

**Conclusions:**

While craniotomy was associated with improved overall survival in the unadjusted analysis, this association was attenuated after PSM, suggesting patient selection may substantially account for the observed difference. Single brain metastasis and radiotherapy exposure were significant independent predictors of survival. Short-term survivor analysis revealed two distinct mortality patterns: the majority died from disease progression, while three patients experienced surgery-related death within 30 days. Rigorous patient selection remains essential in surgical decision-making.

## Highlights

• Craniotomy was associated with improved survival, though this association was attenuated after propensity score matching.
• Single brain metastasis and radiotherapy exposure are significant independent predictors of survival.
• Early mortality reflects heterogeneous causes including both disease progression and surgery-related complications.
• Systemic and neurologic frailty warrant careful surgical candidate selection.

## 1 Introduction

Brain metastases (BM) are the most prevalent tumors occurring within the central nervous system, and their incidence is rising, supported by advancements in imaging techniques and radical progress in cancer treatment (Alexandru et al., 2012; Nayak et al., 2012; Kim and Kim, 2020; Sacks and Rahman, 2020). The spectrum of therapeutic approaches for BM includes systemic treatments, such as targeted agents, radiation therapies, including whole-brain radiation or stereotactic radiosurgery, and surgical options such as craniotomy (Suh et al., 2020; Vogelbaum et al., 2022). Despite these advancements, there are still no sufficient guidelines that help to determine the optimal candidates for craniotomy. The most acknowledged guidelines highlight that patients experience a favorable survival prognosis upon complete resection of all BMs and that surgery can significantly improve the quality of life for those with neurological symptoms (Patchell et al., 1990; Bindal et al., 1993).

Concerning multiple BM, not all lesions qualify for surgery, presenting a significant dilemma for neurosurgeons in deciding which lesions to target for treatment. Currently, Graded Prognostic Assessment (GPA) and algorithms proposed by Hatiboglu et al. have offered valuable guidance (Hatiboglu et al., 2020; Sperduto et al., 2020). GPA incorporates factors, such as age, cancer subtype, Karnofsky Performance Score (KPS), presence of extracranial metastasis, number of BM, and genetic alterations, are used to predict patient survival and aid in surgical decisions (Sperduto et al., 2020). However, while these algorithms are effective in answering “which patients will benefit from surgery,” they offer limited insight into “which patients should avoid surgery” to prevent futile interventions. Although these two questions are closely related, they provide complementary perspectives essential for optimal surgical decision-making. This distinction is particularly critical in intracranial surgery for BM, where surgery-related mortality rates are reported to be higher than those for other brain tumors (Al-Shamy and Sawaya, 2009). This elevated risk is inherently linked to the complex systemic conditions of BM patients, such as advanced cancer stages and various comorbidities, which often complicate postoperative recovery (Byun and Kim, 2023; Lenga et al., 2025). Despite its clinical importance, a detailed analysis of 30-day mortality—especially its direct relationship with surgical intervention in the context of breast cancer BM—has not been sufficiently established, leaving a critical gap in our understanding of early postoperative failure.

Currently, surgery related mortality is defined as mortality occurring within one month after surgery (Sawaya et al., 1998; Lassen et al., 2011). However, cancer patients possess many inherent risks to surgery. Therefore, it is essential to question whether the same definition is appropriate for cancer patients with BM who are potential candidates for craniotomy. In this study, we evaluated the most significant factors affecting survival in patients with breast cancer (BC) with BM and assessed which subgroups could potentially benefit from craniotomy. We also aimed to scrutinize the nature of early postoperative mortality to distinguish between surgery-specific complications and aggressive disease progression, thereby identifying patients who might show unfavorable outcomes despite meeting surgical criteria. This focus on identifying beneficial subgroups aims to refine surgical decision-making and ensure that interventions are strategically targeted and optimally beneficial for patient outcomes.

## 2 Methods

### 2.1 Study population and data collection

This retrospective cohort study focused on patients with BC who were diagnosed with BM via imaging between July 2006 and August 2022. In total, 191 patients were initially recruited for the study. Patients without an electronic medical record (n = 22), those who underwent surgery for BM at another institution (n = 9), or those with a sarcoma diagnosis (n = 3) were excluded, resulting in 157 patients to be included in the final cohort. Clinical and treatment information were extracted from electronic records, including demographics (age at BM diagnosis, sex), cancer subtype (luminal-like, HER2-enriched, triple-negative), KPS, number of BM (categorized as single, oligo-metastasis, or multiple metastases), presence of leptomeningeal seeding (LMS), presence of extracranial metastasis (ECM), and treatment details (chemotherapy, hormone therapy, HER2 targeted therapy, immunotherapy, craniotomy, and radiotherapy). According to the RANO criteria, lesions <10 mm that displayed a nodular pattern and did not have vascular markings were also considered as BM and included in the analysis (Lin et al., 2015). This comprehensive approach aimed to facilitate extensive analysis of survival outcomes across diverse clinical scenarios and treatment modalities.

### 2.2 Surgical indication

All patients were evaluated comprehensively and individually for craniotomy based on their GPA score, KPS, number of BM, and neurological symptoms. The criteria for craniotomy were established according to the methodology outlined in the study by Hatiboglu et al. which served as a reference for selecting suitable surgical candidates (Hatiboglu et al., 2020). In patients with a single BM, surgical treatment was prioritized when there was no ECM and the tumor was not located in an eloquent area. For patients with oligo-BM or multiple BM, surgery was prioritized if neurological symptoms were present, while radiation therapy was preferred for asymptomatic cases. For patients who underwent craniotomy, whole brain radiation therapy (WBRT) was generally considered first, although in some cases, stereotactic radiosurgery (SRS) or fractionated stereotactic radiotherapy (FSRT) was used instead.

### 2.3 Outcome assessment

**Fig. 1:**
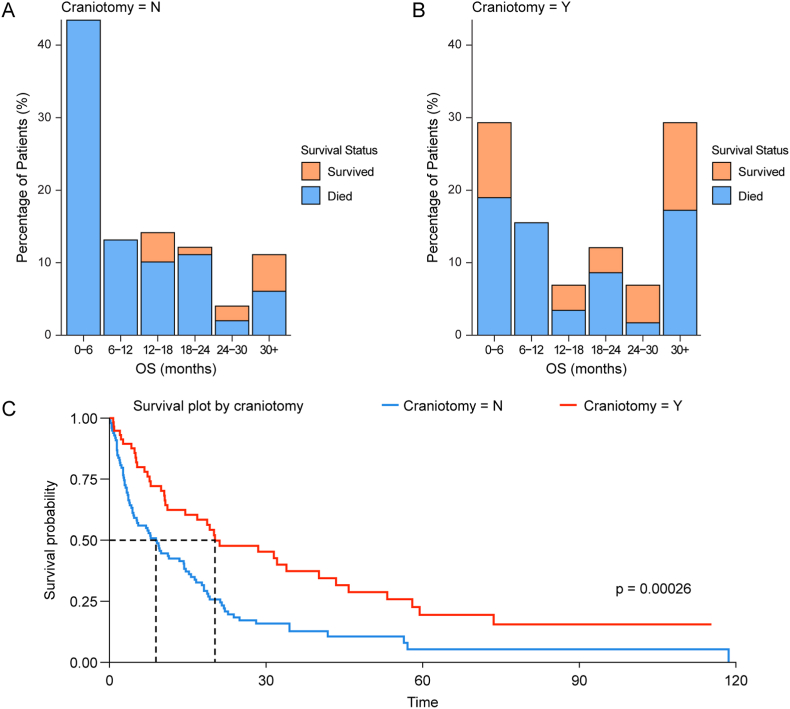
Overall survival of patients with breast cancer brain metastasis. A: Histogram of survival of patients who did not undergo craniotomy for brain metastasis. B: Histogram of survival of patients who underwent craniotomy for brain metastasis. C: Kaplan–Meier survival plot for craniotomy.

The primary focus of the outcome was to evaluate the impact of craniotomy on the survival rate. Initial investigations assessed the influence of craniotomy on overall survival. In this study, we defined overall survival as the time interval the patient survived since the date of initial diagnosis of BM through computed tomography or magnetic resonance imaging. Through Cox regression analysis of variables from the updated breast GPA (Sperduto et al., 2020), significant factors were identified to further explore the survival benefits of craniotomy. Patients whose overall survival was less than 6 months after craniotomy, as shown as the inflection point on the survival plot, were classified as “short survivors” (Fig. 1). For these patients, clinical characteristics were reviewed retrospectively by two doctors independently through electronic medical records to identify peri-operative factors and events that could attribute to the cause of their death. Their explicit cause of death was classified primarily based on their death certificates which was issued by a third attending physician who are not a co-author of this manuscript.

### 2.4 Statistical analysis

Kaplan–Meier analysis was used for overall survival assessment, employing the survival and Survminer R packages for calculation and log-rank score determination. Cox regression analysis, examining factors such as age, number of BM, KPS, cancer subtype, ECM and various modalities of treatment was conducted using the survival package in R. Propensity score matching (PSM) was used to correct the imbalance of items showing differences between the two groups. We implemented a 1:1 nearest neighbor matching, with a caliper width of 0.2 standard deviations of the logit distance measured using the R-package, “MatchIt.” The covariates used for matching included the age at diagnosis of BM, cancer subtypes, number of BM, size and location of the lesion, KPS, LMS, treatment era, radiotherapy type, chemotherapy exposure and extracranial metastasis (Nayak et al., 2012). All analyses were performed using R software version 4.1.3, with p < 0.05 denoting statistical significance, ensuring thorough and meaningful data interpretation.

## 3 Results

### 3.1 Demographics

The patient characteristics are detailed in Table 1. The mean age of the cohort was 52 years, with a balanced distribution across the craniotomy (58 patients) and non-craniotomy (99 patients) groups. The time interval from initial brain metastasis diagnosis to craniotomy was 10.9 days on average. The median overall survival of the cohort was 14.5 months (95% CI, 7.9 to 19.2) and the median follow up interval was 10 months (95% CI, 14.0 to 21.1). Triple-negative breast cancer (TNBC) patients constituted 42.4% of the cohort, while luminal-like subtype patients constituted 33.3%, and HER2-positive patients constituted 24.2%. Notably, patients with single BM were more common in the craniotomy group (55.2%) compared to the non-craniotomy group (14.1%), while multiple BM was significantly more frequent in the non-craniotomy group (p < 0.001). LMS and ECM were significantly lower in the craniotomy group (LMS, p = 0.016; ECM, p < 0.001). Most patients had a KPS of 70 or higher, with better overall KPS scores observed in the craniotomy group, although the difference was not statistically significant (p = 0.063). Chemotherapy was administered to most patients (96.2%) and radiotherapy was also commonly used, with 60.6% receiving WBRT and 6.1% undergoing SRS. PSM was performed for 116 patients. A total of 58 pairs were matched, resulting in 58 patients in the “Craniotomy” and “No craniotomy” groups, while 41 unmatched patients from the control group were excluded. A balanced plot showing the standardized mean differences before and after matching is provided as Supplementary Fig. 1. The demographics of the surgical covariates are included in Supplementary Tables 1 and 2 Within the craniotomy group, more than half of the patients had supratentorial lesion (53.4%) and 25.9% of the patients showed significantly more midline shift on pre-operative imaging (27.6%, p < 0.001).

**Table 1:** Patient demographics before and after propensity score matching.

	Before PSM			ASD	After PSM			ASD
	No craniotomy (n = 99)	Craniotomy (n = 58)	p-value		No craniotomy (n = 58)	Craniotomy (n = 58)	p-value	
Age, mean ± SD (years)	52.3 ± 9.2	52.5 ± 9.3	0.903	0.0199	51.6 ± 9.4	52.5 ± 9.3	0.610	0.0954
Subtype			0.027				0.016	
- HER2	24 (24.2%)	10 (17.2%)		0.1853	13 (22.4%)	10 (17.2%)		0.1369
- luminal like	33 (33.3%)	32 (55.2%)		0.4391	17 (29.3%)	32 (55.2%)		0.5200
- TNBC	42 (42.4%)	16 (27.6%)		0.3320	28 (48.3%)	16 (27.6%)		0.4629
Number of BM, n (%)			<0.001				<0.001	
- single (n = 1)	14 (14.1%)	32 (55.2%)		0.8250	10 (17.2%)	32 (55.2%)		0.7627
- oligo (n = 2,3)	21 (21.2%)	11 (19.0%)		0.0573	15 (25.9%)	11 (19.0%)		0.1759
- multi (n > 3)	64 (64.6%)	15 (25.9%)		0.8857	33 (56.9%)	15 (25.9%)		0.7087
LMS, n (%)	15 (15.2%)	1 (1.7%)	0.016	1.0315	8 (13.8%)	1 (1.7%)	0.037	0.9272
KPS, n (%)			0.063	0.2517			0.187	0.0984
- <70	39 (39.4%)	13 (22.4%)			22 (37.9%)	13 (22.4%)		
− 70-80	54 (54.5%)	38 (65.5%)			31 (53.4%)	38 (65.5%)		
− 90-100	6 (6.1%)	7 (12.1%)			5 (8.6%)	7 (12.1%)		
ECM, n (%)	83 (83.8%)	28 (48.3%)	<0.001	0.7117	45 (77.6%)	28 (48.3%)	0.002	0.5866
Treatment era, n (%)			0.540	0.1039			0.592	0.0391
- before 2003	0	0			0	0		
− 2003-2013	38 (38.4%)	18 (31.0%)			21 (36.2%)	18 (31.0%)		
− 2013-2016	15 (15.2%)	12 (20.7%)			8 (13.8%)	12 (20.7%)		
- after 2016	46 (46.5%)	28 (48.3%)			29 (50.0%)	28 (48.3%)		
Radiotherapy, n (%)			0.222				0.624	
- FSRT	6 (6.1%)	7 (12.1%)		0.1844	3 (5.2%)	7 (12.1%)		0.2117
- SRS	6 (6.1%)	2 (3.4%)		0.1432	2 (3.4%)	2 (3.4%)		0
- WBRT	60 (60.6%)	39 (67.2%)		0.1414	42 (72.4%)	39 (67.2%)		0.1102
Chemotherapy, n (%)	97 (98.0%)	54 (93.1%)	0.268	0.1924	56 (96.6%)	54 (93.1%)	0.675	

ASD, absolute standardized mean difference; PSM, propensity score matching; SMD, standard mean deviation; N, number; SD, standard deviation; HER2, human epidermal growth factor receptor 2; TNBC, triple-negative breast cancer; BM, brain metastasis; LMS, leptomeningeal seeding; KPS, Karnofsky performance score; ECM, extracranial metastasis; FSRT, fractionated stereotactic radiotherapy; SRS, stereotactic radiosurgery; WBRT, whole brain radiation therapy.

### 3.2 Clinical factors impacting survival

In our cohort, 30% of the patients who underwent craniotomy had a better prognosis with a 30-month survival rate that was significantly higher than that of those who did not undergo the procedure, as shown in the Kaplan–Meier survival analysis (p < 0.001; Fig. 1). Univariate Cox regression analysis evaluated known prognostic factors, such as age, subtype, number of BM, KPS, and ECM, revealing significant effects of multiple BM (p < 0.001, p = 0.002), radiotherapy (<0.001, p = 0.008) and craniotomy (p < 0.001, p = 0.001), both before and after propensity score matching (Table 2, Table 3). In the multivariate Cox regression model, the significant factors included oligometastases (p = 0.001, p = 0.001), multiple BM (p < 0.001, p = 0.008), and radiotherapy exposure (p = 0.0069, p = 0.0159; Table 2, Table 3). Although multivariate Cox regression analysis did not show clinical significance of craniotomy, Kaplan-Meier analysis after PSM showed that patients who underwent craniotomy had improved survival compared to those who did not (p < 0.001; Supplementary Fig. 2). To further address potential immortal-time bias, a 30-day landmark analysis was performed, which similarly demonstrated a significant association between craniotomy and survival (p = 0.008; Supplementary Fig. 3). Based on the year in which trastuzumab, pertuzumab and palbociclib was endorsed for use in South Korea (2003, 2013, 2016 respectively), we have subgrouped the patients in the year they were diagnosed with breast cancer and performed separate survival analysis. The results are included as Supplementary Figure 4, 5 and 6.

**Table 2:** Univariate and multivariate Cox regression models for patients’ survival before propensity score matching.

	Univariate Cox regression analysis				Multivariate Cox regression analysis			
	Exp (B)	Lower 95% CI	Upper 95% CI	p-value	Exp (B)	Lower 95% CI	Upper 95% CI	p-value
Age (years)	0.9972	0.9766	1.018	0.794	0.9911	0.9691	1.014	0.4331
Subtype								
HER2	Ref				Ref			
luminal like	1.103	0.6775	1.794	0.6944	1.1563	0.6702	1.995	0.6017
TNBC	1.937	1.1819	3.174	0.009**	0.8120	0.4690	1.406	0.4569
Number of BM								
Single (n = 1)	Ref				Ref			
Oligo (n = 2, 3)	1.637	0.8791	3.047	0.12	2.9650	1.5198	5.784	0.001***
Multiple (n > 3)	2.587	1.7467	3.832	<0.001***	2.7980	1.5360	5.097	<0.001***
KPS								
90–100	Ref				Ref			
70–80	1.4083	0.9546	2.078	0.0844	1.4121	0.9143	2.181	0.1197
<70	0.9499	0.4407	2.048	0.8957	1.4151	0.5978	3.349	0.4297
ECM	1.546	1.022	2.339	0.0391*	0.9908	0.6132	1.601	0.9698
Craniotomy	0.4884	0.3302	0.7225	<0.001***	0.6952	0.4136	1.169	0.1701
Radiotherapy	0.4973	0.3301	0.7492	<0.001***	0.5273	0.3314	0.839	0.0069**
Targeted therapy	0.643	0.4429	0.9336	0.0203*	0.7068	0.4674	1.069	0.1002

Exp (B), exponential value of B; CI, confidence interval; HER2, human epidermal growth factor receptor 2; TNBC, triple-negative breast cancer; BM, brain metastasis; KPS, Karnofsky performance score; ECM, extracranial metastasis.

**Table 3:** Univariate and multivariate Cox regression models for patients’ survival after propensity score matching.

	Univariate Cox regression analysis				Multivariate Cox regression analysis			
	Exp (B)	Lower 95% CI	Upper 95% CI	p-value	Exp (B)	Lower 95% CI	Upper 95% CI	p-value
Age (years)	0.9881	0.9648	1.012	0.328	0.9806	0.9558	1.0061	0.1355
Subtype								
HER2	Ref				Ref			
luminal like	0.7846	0.4178	1.473	0.451	0.8943	0.4551	1.7574	0.7458
TNBC	1.2344	0.6621	2.301	0.508	0.6300	0.3128	1.2690	0.1959
Number of BM								
Single (n = 1)	Ref				Ref			
Oligo (n = 2, 3)	2.781	1.551	4.987	<0.001***	3.4520	1.6432	7.2519	0.001**
Multiple (n > 3)	2.183	1.320	3.610	0.002**	2.5899	1.2795	5.2423	0.008**
KPS								
90–100	Ref				Ref			
70–80	1.3311	0.8366	2.118	0.227	1.4558	0.8416	2.5183	0.1792
<70	0.8451	0.3648	1.958	0.695	1.2370	0.4745	3.2247	0.6634
ECM	1.464	0.9315	2.3	0.0985	1.0732	0.6467	1.7812	0.7845
Craniotomy	0.487	0.316	0.7505	0.001**	0.6574	0.3766	1.1474	0.1399
Radiotherapy	0.5098	0.3092	0.8406	0.008**	0.4846	0.2688	0.8736	0.0159*
Targeted therapy	0.6571	0.427	1.011	0.0563	0.7234	0.4427	1.1822	0.1963

Exp (B), exponential value of B; CI, confidence interval; HER2, human epidermal growth factor receptor 2; TNBC, triple-negative breast cancer; BM, brain metastasis; KPS, Karnofsky performance score; ECM, extracranial met.

### 3.3 Short survivors

From our craniotomy cohort, 16 patients (27.5%) showed less than 6 months survival and were subgrouped as “short survivors”. From the survival plot that compared the overall survival of those who received craniotomy and those who did not, evident discrepancy in the number of patients was shown in the 0-6 months interval (Fig. 1). Among 16 patients, 10 patients (62.4%) were diagnosed with multiple metastasis, and 2 patients (12.5%) were diagnosed with leptomeningeal seeding before surgery. The overall survival of this subgroup was 3.0 ± 1.6 months. Of 16 patients, 12 patients (75.0%) had extracranial metastasis, with 7 patients (43.7%) diagnosed with more than 2 distinct regions in the body. Lung metastasis was observed in 10 patients (62.5%) with pneumonia being the most common primary cause of death (25.0%). Seven patients (43.7%) who were transferred to secondary hospitals for hospice care and died outside our primary research institution were categorized as progression of disease in terms of cause of death (Table 4).

**Table 4:** Demographics of 16 short survivors.

Age, mean ± SD (years)	51.1 ± 11.8
Subtype, n (%)	
- HER2	1 (6.3)
- luminal-like	4 (25.0)
- TNBC	11 (68.8)
Number of BM, n (%)	
- multiple (n > 3)	5 (31.2)
- oligo (n = 2,3)	5 (31.2)
- single (n = 1)	4 (25.0)
LMS, n (%)	2 (12.5)
KPS, n (%)	
<60	2 (12.5)
70–80	11 (68.7)
90–100	3 (18.7)
ECM, n (%)	
- Liver	3 (18.7)
- Lung	10 (62.5)
- Bone	4 (25.0)
- Other	4 (25.0)
OS, mean ± SD (months)	3.0 ± 1.6
Cause of death, n (%)	
- Pneumonia	4 (25.0)
- Increased ICP	3 (18.7)
- Multiorgan failure	1 (6.2)
- Infection	1 (6.2)
- Disease progression	7 (43.7)

Three patients (18.8% within the “short survivor” cohort, 5.2% within the “craniotomy” cohort) died less than 30 days after craniotomy and their preoperative conditions and postoperative progress is summarized in Table 5. Patient #1 was diagnosed radiologically with leptomeningeal seeding and a large sized mass that led to hydrocephalus. The patient showed symptoms of increased intracranial pressure (IICP) prior to surgery. Reduction of mass effect through craniotomy was thought to be favorable in resolving hydrocephalus and its symptoms, but the patient unexpectedly underwent respiratory failure after surgery to resolve the IICP. Patient #3 suffered numerous aspiration events due to postoperative dysphagia. Patient #3 was not radiologically diagnosed with leptomeningeal seeding, but showed CSF pressure of 40cmH2O, evident for IICP. Eventually the patient died of respiratory failure due to aspiration pneumonia. Patient #2 was preoperatively on antibiotics for urinary tract infection prior to surgery. However, the patient underwent multiple organ failures during the recovery phase after surgery leading to death. From our analysis, most of the short survivors died of disease progression while those who have been experiencing IICP or had pre-existing condition died of post-operative complications.

**Table 5:** Pre-operative evaluation and post-operative progress of patients with less than one month survival.

	Breast cancer subtype	Pre-operative evaluation			Cause of death	Postoperative progress
		Neurological symptom	Brain MR	Medical conditions		
Patient #1	TNBC	Headache, nausea with vomiting, cognitive decline	Leptomeningeal seeding, obstructive hydrocephalus due to large cerebellar mass (∼4.0 cm), peritumoral edema	Pleural effusion without lung metastasis	Respiratory failure due to increased ICP	Immediate postoperative vital sign stable and neurology intact → worsened mentality (alert to drowsy) at POD8, with no definite interval change shown by CT scan → Ommaya insertion at POD11 → Respiratory failure with worsened mentality (drowsy to stupor) at POD12, leading to invasive ventilation and ICU care → Cardiac arrest at POD14
Patient #2	TNBC	Dizziness	Multiple metastasis, dominantly in the cerebellum with peritumoral edema (∼2.9 cm)	On broad-spectrum IV antibiotics due to urinary tract infection, high metastatic burden primarily in the lung and liver, chronic kidney disease with electrolyte imbalance	Multiple organ failure (pneumonia, AKI on CKD)	Immediate postoperative vital sign stable and neurology intact → Leukocytosis and CRP elevation at POD 9 despite broad-spectrum antibiotics use prior to surgery→ Severe dyspnea with increasing oxygen demand at POD 18, with chest x-ray showing increased bilateral pleural effusion and atelectasis. AST/ALT elevation was found. → Uncontrollable volume overload state at POD 19 with aggravating kidney function → Unstable vital sign and stuporous mentality leading to cardiac arrest at POD 21
Patient #3	TNBC	Severe headache	Multiple metastasis primarily at basal ganglia (∼3.7 cm) and occipital lobe (∼2.1 cm) with peritumoral edema	None	Aspiration pneumonia due to postoperative cranial neuropathy including dysphagia	Immediate postoperative vital sign stable and neurology intact → Drowsy mentality at POD9 with aspiration event leading to aspiration pneumonia and ICU admission. VFSS showing dysphagia. → Another aspiration event at POD13 and POD 16 leading to re-admission to ICU and invasive ventilation → Spinal tapping showing CSF pressure of 40cmH2O at POD17 → Loss of pupil reflex at POD 22. Cardiac arrest at POD 23

## 4 Discussion

This study aimed to identify subgroups of patients with BC and BM who would benefit from craniotomy and to identify factors that may drive early postoperative mortality. Our findings demonstrate that craniotomy may have association in the candidates selected for surgery via previously reported guideline, even after adjusting for confounding factors. However, this result was only significant before PSM analysis. Among various GPA factors, number of metastases and exposure to radiotherapy was significantly related to survival even after PSM. Notably, detailed analysis of the 16 “short survivor” group revealed that mortality within the first three months was predominantly driven by disease progression rather than surgical complications. Surgery-related mortality was evident in 3 patients, who all died within 2-3 weeks after surgery. These findings suggest that the definition of surgery-related mortality—conventionally limited to 30 days—may need to be reconsidered in the context of surgical decision making, where underlying disease plays a major role in early mortality (Johnson et al., 2002).

The GPA is an important tool for predicting prognosis and providing reliable information through the analysis of large patient data and continuous updates (Sperduto et al., 2020). So far, surgical intervention for metastatic brain tumors has been reported to have survival benefits only for single lesions (Patchell et al., 1990). Retrospective studies have shown that surgical resection can be beneficial in treating patients with BC with central nervous system metastases; however, the results have been typically limited to patients with a single BM, good KPS, and controlled extracranial disease (Ene and Ferguson, 2022). Our PSM analysis reinforces such decisions, as association with survival were evident when clinical variables were adjusted to be balanced.

In determining the overall prognosis after craniotomy, our study shows number of brain metastases and exposure to radiotherapy are the most critical determinants. While previous guidelines have primarily focused on patient performance status or systemic disease control, our analysis identified oligometastases and multiple metastases as independent predictors of poor prognosis. The total disease burden was also a major driver of mortality; among the “short survivors” who died within 6 months of surgery, 75% had multiple intracranial lesions or leptomeningeal seeding. This suggests that while craniotomy may reduce mass effect, the observed association with survival is substantially attenuated in patients with aggressive biology (TNBC) or extensive intracranial burden. Therefore, surgical decision-making should consider the two biological variables over simple anatomical considerations.

Our descriptive analysis of the 16 “short survivors” (survival <6 months) may provide some insight into post-craniotomy mortality. In our cohort, deaths attributable to surgery-related complications were largely confined to the first month post-operation. Among the short survivors, only three patients (18.8%) died within 30 days due to acute complications such as IICP or multi-organ failure. In contrast, the remaining deaths occurring between 1 and 6 months were caused by the rapid progression of systemic disease or intracranial recurrence, rather than the surgical procedure itself. Our results also show that majority of these patients (62%) harbored multiple metastases or extracranial disease, and some presented with leptomeningeal seeding—factors that herald a precipitous decline. This finding is consistent with previous reports indicating that approximately 72% to 78% of deaths in patients with brain metastases are primarily driven by systemic disease progression rather than local intracranial failure (Paek et al., 2005; Niedermeyer et al., 2024). These findings suggest the need for a nuanced discussion regarding the temporal definition of ‘surgery-related mortality'—whether it should be conventionally defined at 1 month or extended to 3 or 6 months in advanced cancer populations (Neervoort et al., 2010; Hadanny et al., 2020; Jimenez et al., 2022; Kreatsoulas et al., 2025). As early mortality in these patients often reflects the aggressive biology of the terminal cancer rather than a failure of the surgical intervention itself, our results of 30-day mortality analysis support the need for considering natural progression of metastatic disease as the cause of death.

The clinical trajectories of the three patients who died within 30 days emphasize the inherent risks of intervening in cases with irreversible physiological damage. For instance, patients suffering from prolonged IICP due to large masses or LMS showed limited recovery despite successful surgical debulking, eventually succumbing to respiratory failure. Furthermore, pre-existing systemic vulnerabilities, such as chronic infections or renal insufficiency, were found to trigger a cascade of multi-organ failure during the critical postoperative recovery phase (Dasenbrock et al., 2017; Khalafallah et al., 2021; Wang et al., 2024). These findings suggest that while craniotomy is an effective palliative tool, its application should be carefully reconsidered for patients who have already crossed a threshold of systemic and neurological frailty.

This study has several limitations. First, as a retrospective single-institution study, the results may be subject to selection bias, as patients undergoing craniotomy were likely to have better performance status than those who did not. However, we attempted to adjust for this bias through PSM analysis. Some variables were not successfully matched even after PSM, possibly due to the large discrepancy in the sample size between the “craniotomy” and “non-craniotomy” group. Second, while we focused on overall survival and perioperative mortality, we did not quantitatively assess quality of life (QOL) or functional independence post-surgery. Previous studies have suggested that QOL can deteriorate in the immediate postoperative period (Nayak et al., 2012; Hatiboglu et al., 2020), a factor that neurosurgeons must discuss with patients. Thirdly, the early mortality analysis is of a very small sample size. With this size of the cohort, identifying the cause of mortality statistically is limited in interpretation. The result of the early mortality analysis should be viewed more as a case series experience and with consecutive studies with more extensive sample size, the result must be validated. Finally, the heterogeneity of systemic treatments received by patients over the long study period (2006–2022) may have influenced survival outcomes. Large-scale, multi-institutional prospective studies are needed to further validate these criteria. Additionally, despite comprehensive propensity score matching, complete covariate balance was not achieved for all variables, particularly for clinically heterogeneous subgroups such as patients with leptomeningeal seeding and non-standard metastatic patterns, which may represent a residual source of confounding. Despite these limitations, this study is expected to make an important contribution to optimizing the candidate selection for surgery in patients BC with BM.

## 5 Conclusion

This study evaluated the survival outcomes of craniotomy in breast cancer patients with brain metastases. While craniotomy was associated with improved overall survival in the unadjusted analysis, this association was attenuated after propensity score matching, suggesting that patient selection characteristics may substantially account for the observed survival difference. Single brain metastasis and radiotherapy exposure were identified as significant independent predictors of survival. Analysis of short-term survivors revealed two distinct patterns of early mortality: the majority died from systemic disease progression, while three patients experienced surgery-related death within 30 days. Given the small sample size, these findings should be interpreted with caution, but underscore the importance of rigorous patient selection in surgical decision-making for this population.

## Ethics approval

This is an observational study. Approval was granted by the institutional review board of Gangnam Severance Hospital (IRB no: 3-2026-0122). Patient consent was waived as the data were only analyzed in an anonymized form.

## Author contributions

J.Y. and J.O. contributed to the study conception and design. Material preparation, data collection analysis were performed by S.J.A., H.H.P. J.O. and J.Y. Analyses were performed by J.L., S.B.L. and J.Y. The first draft of the manuscript was written by S.B.L. and all authors commented on previous versions of the manuscript. All authors read and approved the final manuscript.

## Data availability

The datasets generated during and/or analyzed during the current study are available from the corresponding author on reasonable request.

## Declaration of generative AI and AI-assisted technologies in the writing process

Statement:During the preparation of this work the authors used ChatGPT and Gemini in order to improve the readability. After using this tool, the authors reviewed and edited the content as needed and take full responsibility for the content of the manuscript.

## Funding

This research was supported by a 10.13039/501100003725National Research Foundation of Korea (NRF) grant funded by the Korean government (grant number: RS-2023-00246346). Also, this research was funded by a new faculty research seed money grant (2025-32-0033) and a faculty research grant (6-2025-0153) from 10.13039/501100008005Yonsei University College of Medicine, and by the Future Research Grant (D-2025-0001) from Gangnam Severance Hospital, 10.13039/501100008005Yonsei University College of Medicine. The funders had no role in study design, data collection and analysis, decision to publish, or preparation of the manuscript.

## Competing interest

There is no conflict of interest.

## References

[bib1] Al-Shamy G., Sawaya R. (2009). Management of brain metastases: the indispensable role of surgery. J. Neuro Oncol..

[bib2] Alexandru D., Bota D.A., Linskey M.E. (2012). Epidemiology of central nervous system metastases. Prog. Neurol. Surg..

[bib3] Bindal R.K., Sawaya R., Leavens M.E., Lee J.J. (1993). Surgical treatment of multiple brain metastases. J. Neurosurg..

[bib4] Byun J., Kim J.H. (2023). Revisiting the role of surgical resection for brain metastasis. Brain Tumor Res Treat.

[bib5] Dasenbrock H.H., Yan S.C., Smith T.R., Valdes P.A., Gormley W.B., Claus E.B., Dunn I.F. (2017). Readmission after craniotomy for tumor: a national surgical quality improvement program analysis. Neurosurgery.

[bib6] Ene C.I., Ferguson S.D. (2022). Surgical management of brain metastasis: challenges and nuances. Front. Oncol..

[bib7] Hadanny A., Tzubery S., Hadelsberg U., Gonen L., Margalit N. (2020). The outcome of intracranial meningioma surgery in octogenarians: matched cohort study. World Neurosurg..

[bib8] Hatiboglu M.A., Akdur K., Sawaya R. (2020). Neurosurgical management of patients with brain metastasis. Neurosurg. Rev..

[bib9] Jimenez A.E., Cicalese K.V., Chakravarti S., Porras J.L., Azad T.D., Jackson C.M., Gallia G.L., Bettegowda C., Weingart J., Mukherjee D. (2022). Social determinants of health and the prediction of 90-day mortality among brain tumor patients. J. Neurosurg..

[bib10] Johnson M.L., Gordon H.S., Petersen N.J., Wray N.P., Shroyer A.L., Grover F.L., Geraci J.M. (2002). Effect of definition of mortality on hospital profiles. Med Care.

[bib11] Khalafallah A.M., Huq S., Jimenez A.E., Brem H., Mukherjee D. (2021). The 5-factor modified frailty index: an effective predictor of mortality in brain tumor patients. J. Neurosurg..

[bib12] Kim J.S., Kim I.A. (2020). Evolving treatment strategies of brain metastases from breast cancer: current status and future direction. Ther. Adv. Med. Oncol..

[bib13] Kreatsoulas D.C., Kim J., Damante M., Orr A., Wang J., Vignolles-Jeong J., Gruber M., Shah V., Musgrave N., Lonser R., Prevedello D., Elder J.B., Hardesty D.A. (2025). A novel lesion severity index to predict 90-day postoperative survival in brain metastasis patients. J. Neuro Oncol..

[bib14] Lassen B., Helseth E., Ronning P., Scheie D., Johannesen T.B., Maehlen J., Langmoen I.A., Meling T.R. (2011). Surgical mortality at 30 days and complications leading to recraniotomy in 2630 consecutive craniotomies for intracranial tumors. Neurosurgery.

[bib15] Lenga P., Scherer M., Kleineidam H., Unterberg A., Krieg S.M., Dao Trong P. (2025). Neurosurgical management of brain metastases in the elderly: a prospective study on adverse event prevalence and predictors. Neurosurg. Rev..

[bib16] Lin N.U., Lee E.Q., Aoyama H., Barani I.J., Barboriak D.P., Baumert B.G., Bendszus M., Brown P.D., Camidge D.R., Chang S.M., Dancey J., de Vries E.G., Gaspar L.E., Harris G.J., Hodi F.S., Kalkanis S.N., Linskey M.E., Macdonald D.R., Margolin K., Mehta M.P., Schiff D., Soffietti R., Suh J.H., van den Bent M.J., Vogelbaum M.A., Wen P.Y., Response Assessment in Neuro-Oncology, g (2015). Response assessment criteria for brain metastases: proposal from the RANO group. Lancet Oncol..

[bib17] Nayak L., Lee E.Q., Wen P.Y. (2012). Epidemiology of brain metastases. Curr. Oncol. Rep..

[bib18] Neervoort F.W., Van Ouwerkerk W.J., Folkersma H., Kaspers G.J., Vandertop W.P. (2010). Surgical morbidity and mortality of pediatric brain tumors: a single center audit. Childs Nerv. Syst..

[bib19] Niedermeyer S., Schmutzer-Sondergeld M., Weller J., Katzendobler S., Kirchleitner S., Forbrig R., Harter P.N., Baumgarten L.V., Schichor C., Stoecklein V., Thon N. (2024). Neurosurgical resection of multiple brain metastases: outcomes, complications, and survival rates in a retrospective analysis. J. Neuro Oncol..

[bib20] Paek S.H., Audu P.B., Sperling M.R., Cho J., Andrews D.W. (2005). Reevaluation of surgery for the treatment of brain metastases: review of 208 patients with single or multiple brain metastases treated at one institution with modern neurosurgical techniques. Neurosurgery.

[bib21] Patchell R.A., Tibbs P.A., Walsh J.W., Dempsey R.J., Maruyama Y., Kryscio R.J., Markesbery W.R., Macdonald J.S., Young B. (1990). A randomized trial of surgery in the treatment of single metastases to the brain. N. Engl. J. Med..

[bib22] Sacks P., Rahman M. (2020). Epidemiology of brain metastases. Neurosurg. Clin..

[bib23] Sawaya R., Hammoud M., Schoppa D., Hess K.R., Wu S.Z., Shi W.M., Wildrick D.M. (1998). Neurosurgical outcomes in a modern series of 400 craniotomies for treatment of parenchymal tumors. Neurosurgery.

[bib24] Sperduto P.W., Mesko S., Li J., Cagney D., Aizer A., Lin N.U., Nesbit E., Kruser T.J., Chan J., Braunstein S., Lee J., Kirkpatrick J.P., Breen W., Brown P.D., Shi D., Shih H.A., Soliman H., Sahgal A., Shanley R., Sperduto W., Lou E., Everett A., Boggs D.H., Masucci L., Roberge D., Remick J., Plichta K., Buatti J.M., Jain S., Gaspar L.E., Wu C.C., Wang T.J.C., Bryant J., Chuong M., Yu J., Chiang V., Nakano T., Aoyama H., Mehta M.P. (2020). Beyond an updated graded prognostic assessment (Breast GPA): a prognostic index and trends in treatment and survival in breast cancer brain metastases from 1985 to today. Int. J. Radiat. Oncol. Biol. Phys..

[bib25] Suh J.H., Kotecha R., Chao S.T., Ahluwalia M.S., Sahgal A., Chang E.L. (2020). Current approaches to the management of brain metastases. Nat. Rev. Clin. Oncol..

[bib26] Vogelbaum M.A., Brown P.D., Messersmith H., Brastianos P.K., Burri S., Cahill D., Dunn I.F., Gaspar L.E., Gatson N.T.N., Gondi V., Jordan J.T., Lassman A.B., Maues J., Mohile N., Redjal N., Stevens G., Sulman E., van den Bent M., Wallace H.J., Weinberg J.S., Zadeh G., Schiff D. (2022). Treatment for brain metastases: ASCO-SNO-ASTRO guideline. J. Clin. Oncol..

[bib27] Wang P., Zhang Y., Xu W., Zheng Y., Jia L., He J., He M., Chen L., Hao P., Xiao Y., Peng L., Chong W., Hai Y., You C., Fang F. (2024). Association between elevated preoperative red cell distribution width and mortality after brain tumor craniotomy. Neurosurg. Rev..

