# Supplementary material for: Clinical outcomes and survival benefits of craniotomy in breast cancer patients with brain metastases: Focusing on candidate selection and early mortality
Source: Brain Spine. 2026 Jul 7;6:106158. doi: 10.1016/j.bas.2026.106158 (PMC13356780; doi:10.1016/j.bas.2026.106158)

Supplementary Table 1. Demographics of surgical covariates before PSM

| **Covariates** | **No craniotomy (N=99)** | **Craniotomy (N=58)** | **P-value** |
| --- | --- | --- | --- |
| Lesion size |  |  | <0.001 |
| - < 3cm | 32 (32.3 %) | 29 (50.0 %) |  |
| - 3cm or larger | 11 (11.1 %) | 29 (50.0 %) |  |
| - Unmeasurable | 56 (56.6 %) | 0 |  |
| Location |  |  | <0.001 |
| - Supratentorial | 21 (21.2 %) | 31 (53.4 %) |  |
| - Infratentorial | 6 (6.1 %) | 16 (27.6 %) |  |
| - Both | 14 (14.1 %) | 10 (17.2 %) |  |
| - Other (LMS etc.) | 21 (21.2 %) | 1 (1.7 %) |  |
| Posterior fossa tumor | 29 (29.3 %) | 23 (39.7 %) | 0.248 |
| Midline shift | 6 (6.1 %) | 15 (25.9 %) | < 0.001 |
| Hydrocephalus | 5 (5.1 %) | 4 (6.9 %) | 0.901 |
| Preoperative neurology | 90 (90.9 %) | 55 (94.8 %) | 0.561 |
| Urgency | 9 (9.1 %) | 51 (87.9 %) | < 0.001 |
| Extent of resection |  |  | < 0.001 |
| - GTR | 0 | 48 (82.8 %) |  |
| - STR | 0 | 10 (17.2 %) |  |
| Postoperative complication |  |  | 0.031 |
| - Hydrocephalus | 0 | 2 (3.4 %) |  |
| - Motor weakness | 0 | 1 (1.7 %) |  |
| - Postoperative hematoma | 0 | 2 (3.4 %) |  |
| - Cranial nerve palsy | 0 | 1 (1.7 %) |  |
| - None | 0 | 52 (89.7 %) |  |
| Reoperation | 0 | 4 (6.9 %) | 0.034 |

Supplementary Table 2. Demographics of surgical covariates after PSM

| **Covariates** | **No craniotomy (N=58)** | **Craniotomy (N=58)** | **P-value** |
| --- | --- | --- | --- |
| Lesion size |  |  | <0.001 |
| - < 3cm | 28 (48.3 %) | 29 (50.0 %) |  |
| - 3cm or larger | 11 (19.0 %) | 29 (50.0 %) |  |
| - Unmeasurable | 19 (32.8 %) | 0 |  |
| Location |  |  | <0.001 |
| - Supratentorial | 18 (31.0 %) | 31 (53.4 %) |  |
| - Infratentorial | 5 (8.6 %) | 16 (27.6 %) |  |
| - Both | 14 (24.1 %) | 10 (17.2 %) |  |
| - Other (LMS etc.) | 21 (36.2 %) | 1 (1.7 %) |  |
| Posterior fossa tumor | 23 (39.7 %) | 23 (39.7 %) | 1.000 |
| Midline shift | 5 (8.6 %) | 15 (25.9 %) | 0.027 |
| Hydrocephalus | 4 (6.9 %) | 4 (6.9 %) | 1.000 |
| Preoperative neurology | 52 (89.7 %) | 55 (94.8 %) | 0.488 |
| Urgency | 7 (12.1 %) | 51 (87.9 %) | < 0.001 |
| Extent of resection |  |  | < 0.001 |
| - GTR | 0 | 48 (82.8 %) |  |
| - STR | 0 | 10 (17.2 %) |  |
| Postoperative complication |  |  | 0.176 |
| - Hydrocephalus | 0 | 2 (3.4 %) |  |
| - Motor weakness | 0 | 1 (1.7 %) |  |
| - Postoperative hematoma | 0 | 2 (3.4 %) |  |
| - Cranial nerve palsy | 0 | 1 (1.7 %) |  |
| - None | 0 | 52 (89.7 %) |  |
| Reoperation | 0 | 4 (6.9 %) | 0.127 |

Supplementary Figure 1. Balanced plot showing absolute standardized mean differences between the matched and unmatched cohort


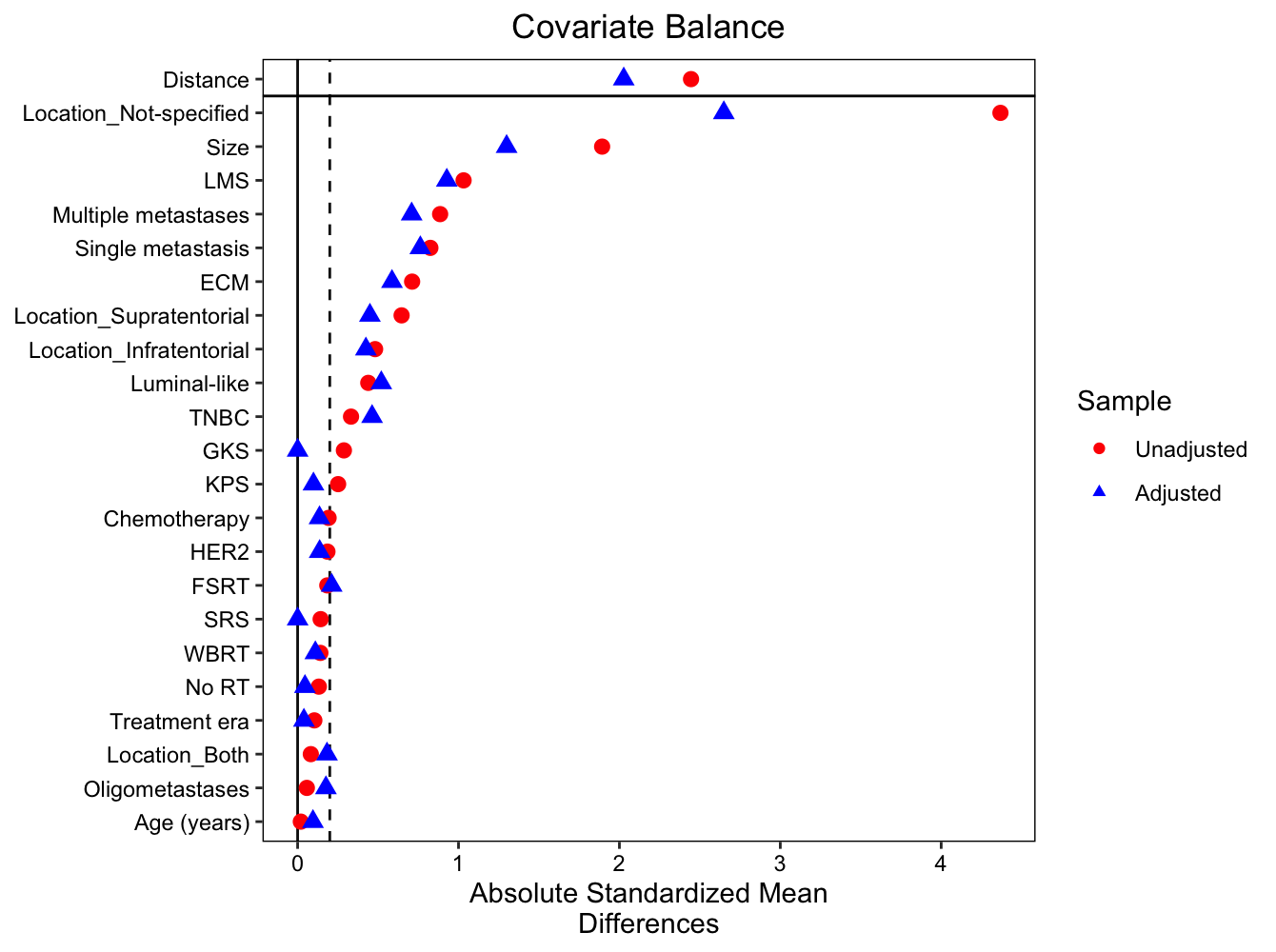


Supplementary Figure 2. Kaplan-Meier survival curves comparing overall survival by craniotomy status after propensity score matching.


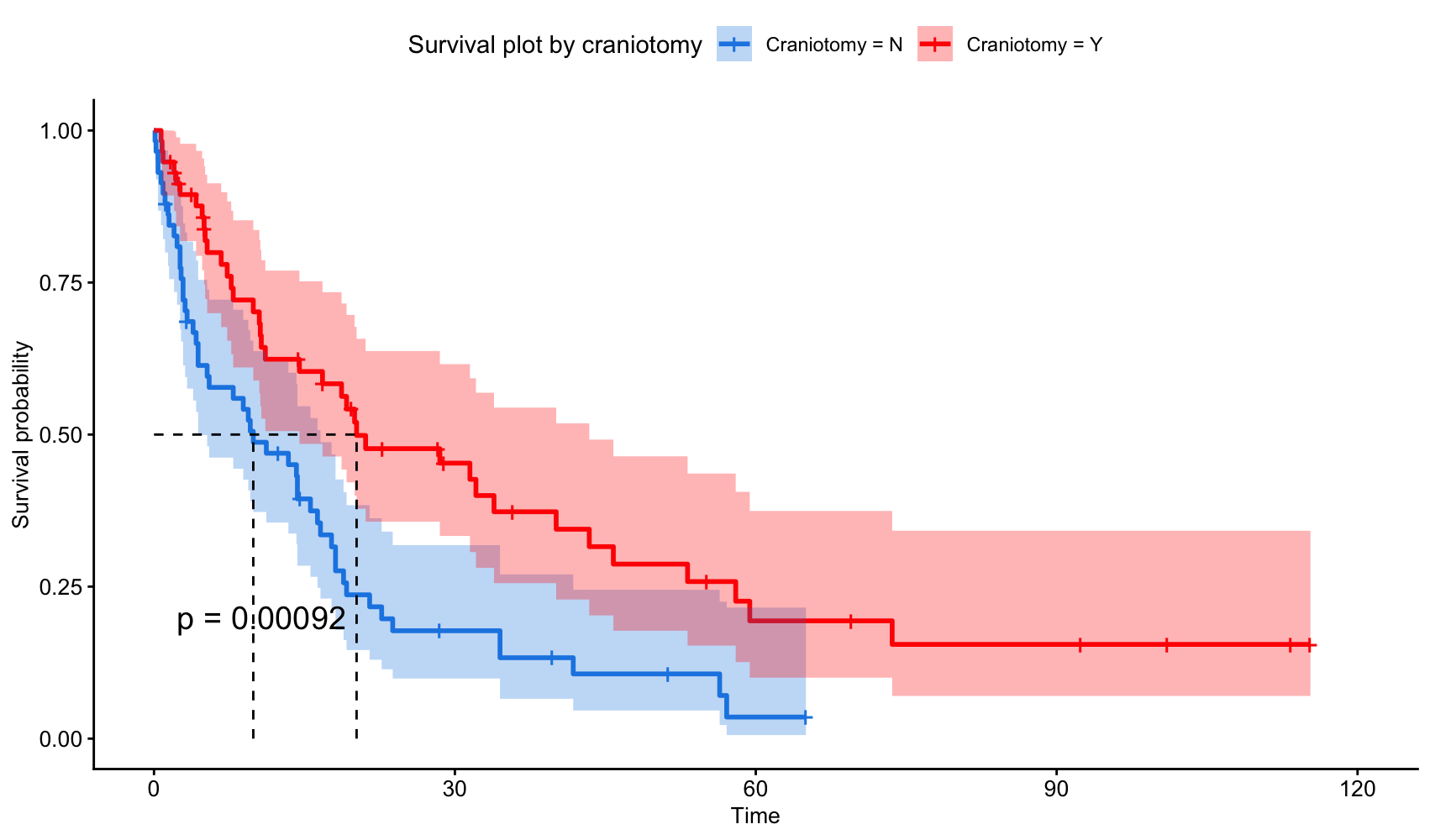


Supplementary Figure 3. Kaplan-Meier survival curves comparing overall survival by craniotomy status with 30-day landmark analysis.


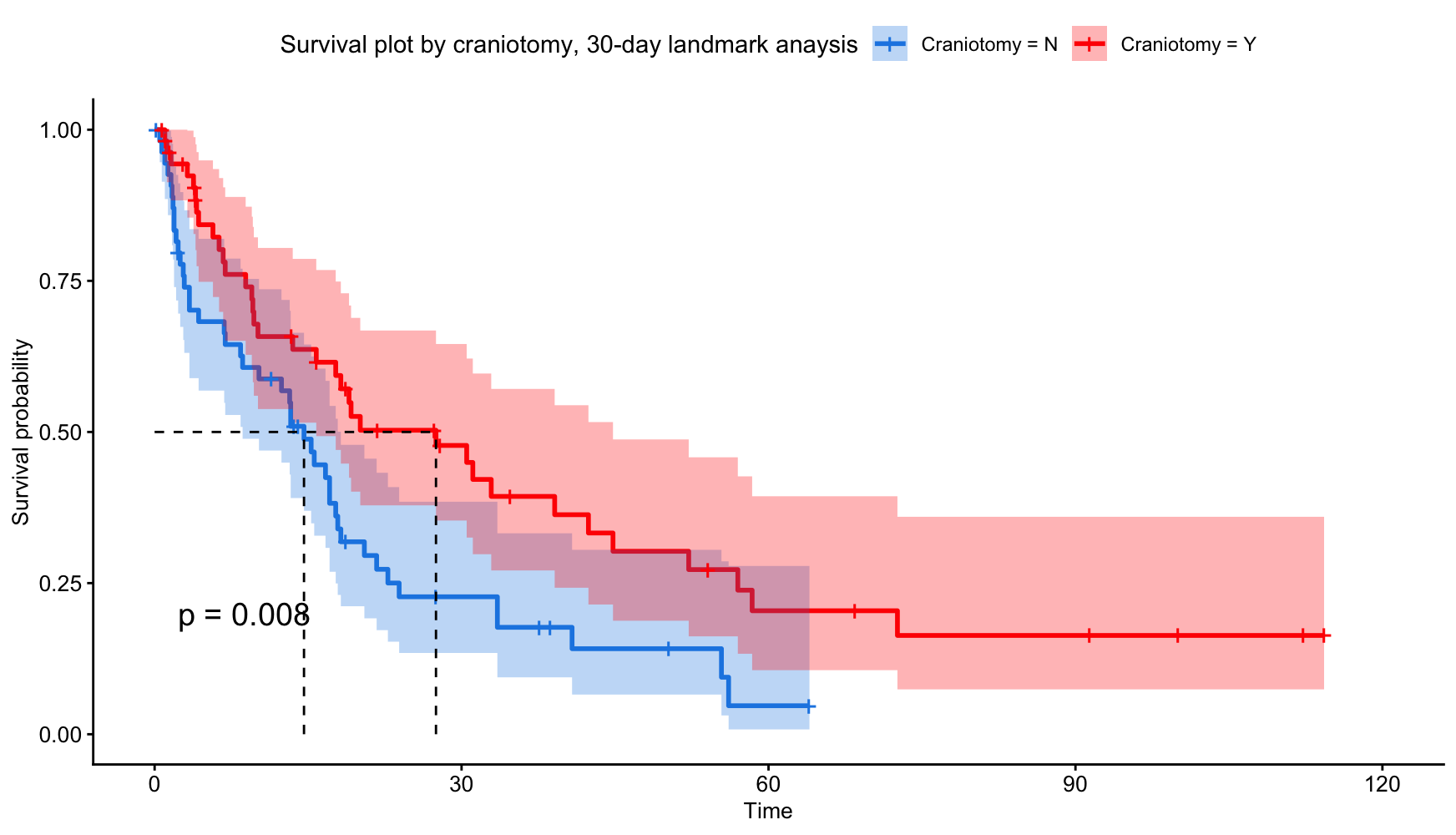


Supplementary Figure 4. Kaplan-Meier survival plot comparing patients who received craniotomy and those that did not during the year 2003-2013, with adjustment using propensity score matching


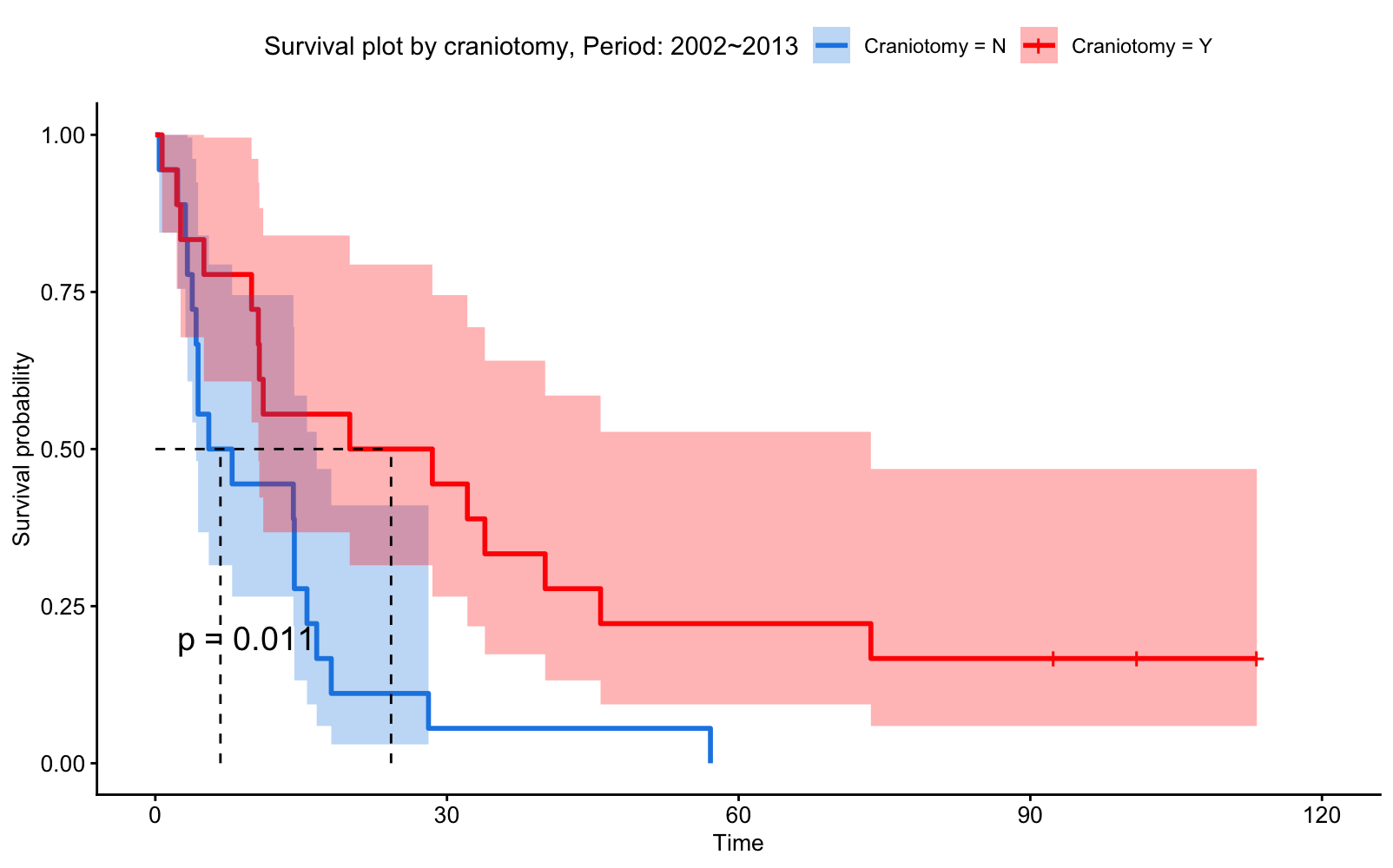


Supplementary Figure 5. Kaplan-Meier survival plot comparing patients who received craniotomy and those that did not during the year 2013-2016, with adjustment using propensity score matching


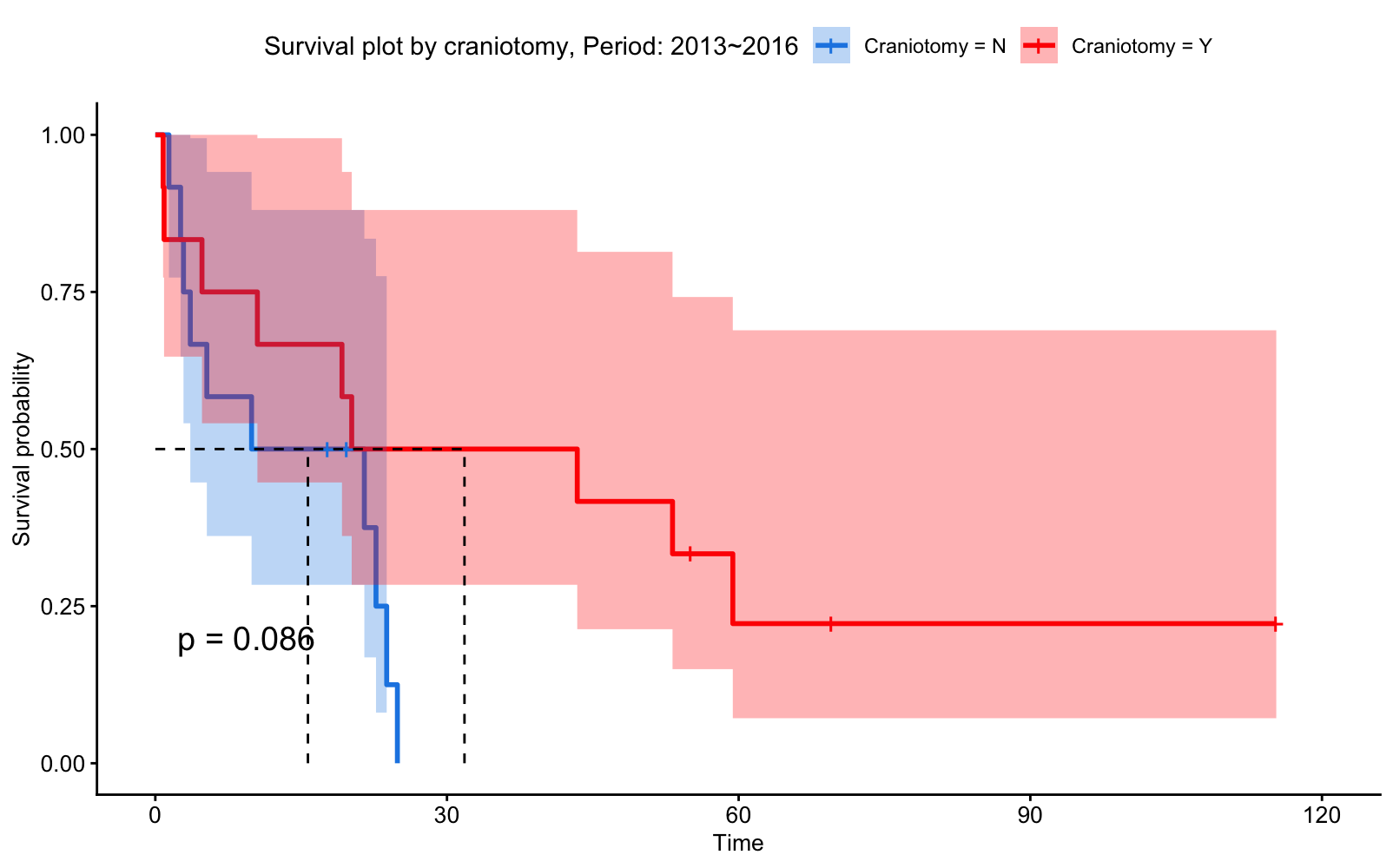


Supplementary Figure 6. Kaplan-Meier survival plot comparing patients who received craniotomy and those that did not on the year after 2016, with adjustment using propensity score matching


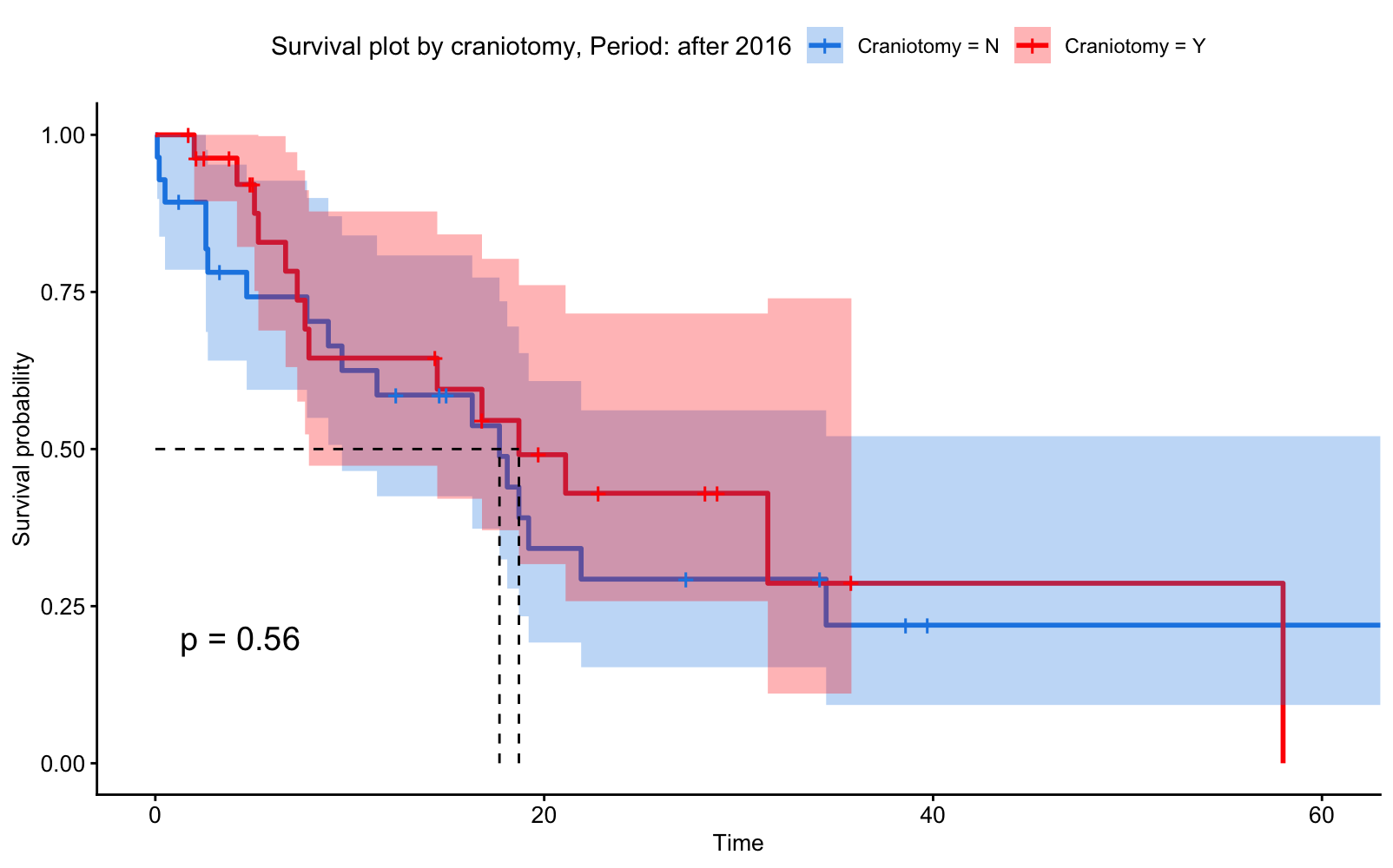

Supplement: Multimedia component 1 [file mmc1.docx]
